# TGFβ-induced cytoskeletal remodeling mediates elevation of cell stiffness and invasiveness in NSCLC

**DOI:** 10.1038/s41598-019-43409-x

**Published:** 2019-05-21

**Authors:** E. Gladilin, S. Ohse, M. Boerries, H. Busch, C. Xu, M. Schneider, M. Meister, R. Eils

**Affiliations:** 1 German Cancer Research Center, Div. Bioinformatics and Omics Data Analytics, Mathematikon - Berliner Str. 41, 69120 Heidelberg, Germany; 2 University Heidelberg, BioQuant, Im Neuenheimer Feld 267, 69120 Heidelberg, Germany; 3 University of Freiburg, Institute of Molecular Medicine and Cell Research (IMMZ), Stefan-Meier-Str. 17, 79104 Freiburg, Germany; 4 University of Lübeck, Institute of Experimental Dermatology, Ratzeburger Allee 160, 23538 Lübeck, Germany; 5 Thoraxklinik at Heidelberg University Hospital, Amalienstr. 5, 69126 Heidelberg, Germany; 6 German Cancer Consortium (DKTK), German Cancer Research Center (DKFZ), Heidelberg, Germany; 7 Translational Lung Research Center Heidelberg (TLRC-H), Member of the German Center for Lung Research (DZL), Heidelberg, Germany; 8 Center for Digital Health, Berlin Institute of Health, and Charité Universitätsmedizin Berlin, Kapelle-Ufer 2, 10117 Berlin, Germany; 9 Health Data Science Unit, Heidelberg University Hospital, Im Neuenheimer Feld 267, 69120 Heidelberg, Germany; 10 Department for Biometry, Epidemiology and Medical Bioinformatics and Comprehensive Cancer Center Freiburg (CCCF), University Medical Center Freiburg, Faculty of Medicine, University of Freiburg, Breisacherstrasse 153, 79110 Freiburg, Germany; 11 Present Address: Leibniz Institute of Plant Genetics and Crop Plant Research, OT Gatersleben Corrensstrasse 3, 06466 Seeland, Germany

**Keywords:** Non-small-cell lung cancer, Motor proteins

## Abstract

Importance of growth factor (GF) signaling in cancer progression is widely acknowledged. Transforming growth factor beta (TGFβ) is known to play a key role in epithelial-to-mesenchymal transition (EMT) and metastatic cell transformation that are characterized by alterations in cell mechanical architecture and behavior towards a more robust and motile single cell phenotype. However, mechanisms mediating cancer type specific enhancement of cell mechanical phenotype in response to TGFβ remain poorly understood. Here, we combine high-throughput mechanical cell phenotyping, microarray analysis and gene-silencing to dissect cytoskeletal mediators of TGFβ-induced changes in mechanical properties of on-small-cell lung carcinoma (NSCLC) cells. Our experimental results show that elevation of rigidity and invasiveness of TGFβ-stimulated NSCLC cells correlates with upregulation of several cytoskeletal and motor proteins including vimentin, a canonical marker of EMT, and less-known unconventional myosins. Selective probing of gene-silenced cells lead to identification of unconventional myosin MYH15 as a novel mediator of elevated cell rigidity and invasiveness in TGFβ-stimulated NSCLC cells. Our experimental results provide insights into TGFβ-induced cytoskeletal remodeling of NSCLC cells and suggest that mediators of elevated cell stiffness and migratory activity such as unconventional cytoskeletal and motor proteins may represent promising pharmaceutical targets for restraining invasive spread of lung cancer.

## Introduction

Non-small-cell lung adenocarcinoma (NSCLC) is the leading cause of cancer-related mortalities worldwide^1^. Asymptomatic progression, metastasis independent of tumor size and therapy resistance are the major reasons of poor prognosis and treatment failure^2^. Despite recent advances in molecular diagnosis and targeted therapies the 5-year-survival remains at 16%, only^1^. Therefore, a better understanding of cell biological processes responsible for early metastasis and progression is crucial for designing of future therapeutic interventions in lung cancer.

Metastasis is a multistep process that starts with cells from the primary tumor site acquiring an invasive phenotype, invading adjacent healthy tissues, entering the lymphatic or blood vessels and finally leaving them and colonizing new distant sites. Invading cancer cells can employ two very distinct variants of single-cell invasion into surrounding 3D matrix: mesenchymal and amoeboid invasion^3^. Cells that utilize a mesenchymal invasion mode usually have undergone epithelial-to-mesenchymal transition (EMT) and therefore possess fibroblast-like spindle-shaped morphology^4^. These cells have multiple stress fibers, a reorganized network of intermediate filaments as well as well-defined filo- and/or lamelipodia present on their leading edge^5,6^. When invading into the surrounding tissue they actively remodel and degrade the extracellular matrix (ECM) by secreting extracellular matrix metalloproteinases (MMPs)^7^. In contrast, cells with amoeboid invasion are highly deformable, lack well-defined actin stress fibers and proteolytic activity^8^. Instead they rely on membrane blebbing – a process of formation of bubble-like short-lived membrane protrusion^9^. Such cells perform 3D matrix invasion by squeezing into the gaps in ECM rather than degrading it^10^. Growth factors (GF) can induce both types of single cell invasion. Pro-tumorigenic role of GF-dependent signaling have been widely reported^11^. Transforming Growth Factor β (TGFβ) is the most described inducer of EMT, while other ligands such as Epidermal Growth Factor (EGF), Hepatocyte Growth Factor (HGF), Fibroblast Growth Factor (FGF) and Wnt were also reported to cause EMT in certain cell types^12–15^. On the other hand, HGF-stimulation was also reported to cause amoeboid invasion of cancer cells^16^. Co-stimulation of cells with TGFβ and HGF/EGF was previously shown to augment effects of TGFβ-triggered EMT^17,18^.

In the context of lung cancer, TGFβ and HGF are of major interest as activators of various downstream signaling cascades inducing survival as well as mitogenic and migratory responses^19,20^. Expression of the HGF receptor Met is strongly up-regulated in 25% of NSCLC patients and correlates with decreased overall survival^21,22^. TGFβ is a cytokine that is known for its ‘double-edge sword’ role in cancerogenesis: inhibiting tumor development during early stages and providing tumorigenic advantages later on^23^. Elevated tissue levels of TGFβ in lung cancer patients were found to correlate with tumor progression and metastasis rates^24^. Multiple studies demonstrated that these GFs enhance migratory and invasive potential of cancer cells and, thus, increase rates of metastasis^25,26^. However, molecular mechanisms mediating alteration of cell constitutive properties and migratory activity in NSCLC are not yet well understood.

Cytoskeleton structure and cellular mechanical properties are heavily affected in both, amoeboid and mesenchymal, invasion modes^27,28^. However, until now the interrelationship between GF-induced molecular-genetic reprogramming and changes in cell mechanical properties in lung cancer are poorly understood. In this study, we aimed to quantitatively assess alterations in the mechanical phenotype and gene expression of stable and primary NSCLC cell lines in response to single treatment and co-stimulation with the growth factors TGFβ and HGF. For this purpose, a combined approach based on high-throughput mechanical cell probing, cell migration screening, microarray analysis and gene-silencing was applied. Our findings provide a comprehensive picture of phenotypic effects and genomic response of NSCLC cells to TGFβ- and HGF-stimulation that sheds light on the mechanisms of GF-induced invasive tumor spread. In particular, our experimental results show that both growth factors do not affect the G/F-actin ratio but rather crosslinking of actin cytoskeleton. The strongest impact on rigidity and invasiveness of NSCLC cells is observed in response to TGFβ stimulation which induces a large-scale rearrangement of cell mechanical architecture, including overexpression of vimentin intermediate filaments, adhesion/migration relevant proteins and unconventional myosins.

## Results

**Figure 1:**
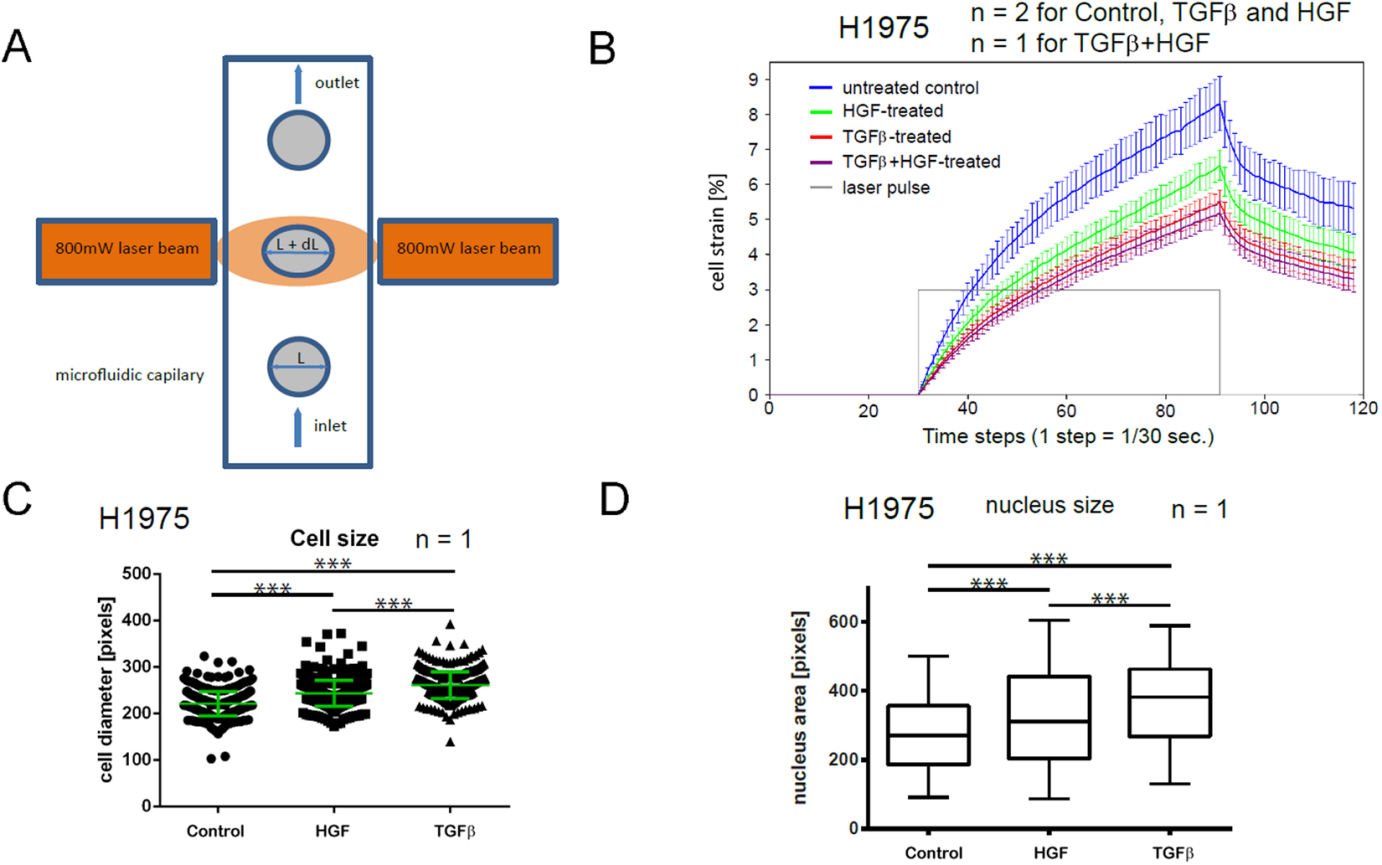
Effects of TGFβ- and HGF-stimulation on rigidity and morphology of NSCLC cells. (**A**) Working principle of the cell optical stretcher: uniaxial cell elongation from the inital diameter *L* to *L*′ = *L* + *dL* under the impact of optical forces. (**B**) Creep-and-recovery curves of untreated, HGF-, TGFβ-treated and co-stimulated H1975 cells. Solid lines show the mean cell strain of bootstrap sample means. Error bars indicate 95% confidence interval which is given by two-fold standard deviation of bootstrap sample means. Cells were treated with 2 ng/ml TGFβ, or 80 ng/ml HGF or combination of both for 24 h in growth factor-depleted medium. Trypsinized cells were injected into the microfluidic system of cell optical stretcher. At least 300 cells per condition were measured. (**C**) Growth factor treatment leads to the increase of cell size of H1975 cells. Cells were treated with 2 ng/ml TGFβ, or 80 ng/ml HGF or left untreated for 24 h in growth factor-depleted medium, trypsinized and measured on cell optical stretcher. Cell diameter prior to laser-induced cell stretching was measured and compared between the conditions. (**D**) H1975 cells were seeded on 24-well plate, treated with 2 ng/ml TGFβ, or 80 ng/ml HGF or left untreated for 24 h in growth factor-depleted medium, stained with Hoechst and imaged with a wide-field fluorescence microscope (Olympus). ImageJ was used to quantify the nuclei area. Center lines show the medians; box limits indicate the 25th and 75th percentiles; whiskers extend 1.5 times the interquartile range from the 25th and 75th percentiles. ***p < 0.001 according to Mann–Whitney test. **n** indicates the number of independent repetitions.

To assess effects of TGFβ- and HGF-stimulation alone and in combination on the mechanical properties of H1975 NSCLC cells in a high-throughput manner, measurements of cell strain response, the so-called creep-and-recovery curves, were performed using the microfluidic optical stretcher (MOS), Fig. 1A. Our experimental results reveal elevation of cell rigidity, i.e., reduction of the maximum cell strain, in GF-treated cell samples in comparison to untreated control by 21% (HGF), 34% (TGFβ) and 37% (TGFβ + HGF), Fig. 1B. Furthermore, a statistically significant increase in size (i.e., cross section area) of unloaded cells and their nuclei upon HGF- and TGFβ-treatment was detected, Fig. 1C,D.

**Figure 2:**
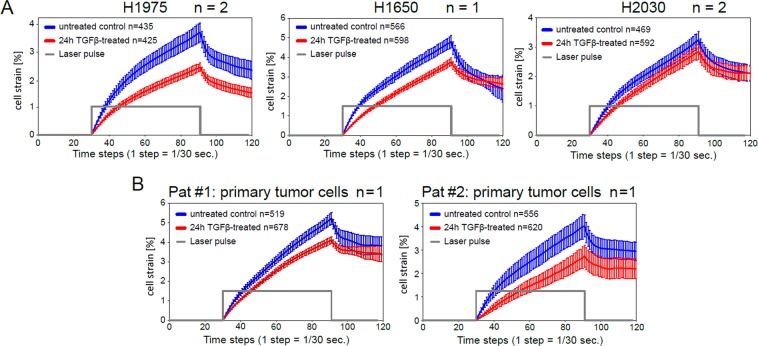
TGFβ-stimulation elevates rigidity of stable and primary NSCLC cell lines. Creep-and-recovery curves of untreated and TGFβ-stimulated H1975, H1650, H2030 cell lines (**A**) and primary lung adenocarcinoma cells derived from two donors (**B**). Cells were treated with 2 ng/ml TGFβ, or left untreated in growth factor-depleted medium for 24 h. Trypsinized cells were injected into the microfluidic system of cell optical stretcher. **n** inside the boxes indicates the number of cells measured per condition, **n** outside the boxes corresponds to the number of independent repetitions.

As TGFβ showed the strongest impact on stiffness of H1975 cells, MOS measurements of cell compliance were extended to two additional NSCLC cell lines (H1650, H2030) as well as primary lung adenocarcinoma cells derived from two donors. All stable and primary NSCLC cell lines displayed the same effect of cell stiffening upon TGFβ-stimulation, Fig. 2. Interestingly, the epithelial NSCLC cell lines (H1975, H1650) exhibited a stronger increase in cell rigidity in comparison to the mesenchymal NSCLC cell line (H2030).

**Figure 3:**
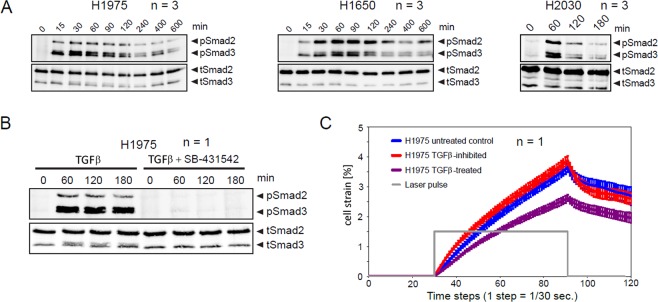
TGFβ/Smad signaling pathway activation in response to TGFβ-stimulation. H1975, H1650 and H2030 cells were kept in serum-free medium overnight. Cells were stimulated with 2 ng/ml TGFβ and lysed at the indicated time points. Subsequently, Smad2/3 immunoprecipitation was followed by immunoblotting. (**A**) Immunoblotting data of H1975, H1650 and H2030 cells. One representative example is shown. (**B**) Treatment with TGFβR inhibitor SB-431542 (10 μM) completely abolishes Smad2/3 phosphorylation upon TGFβ-treatment. (**C**) TGFβ-induced reduction of cell deformability is abolished upon application of TGFβR inhibitor. H1975 cells were treated with 2 ng/ml TGFβ, in presence or absence of 10 μM SB-431542 inhibitor for 24 h. Trypsinized cells were injected into the microfluidic system of cell optical stretcher. At least 300 cells per condition were measured. **n** indicates number of independent repetitions.

To prove that alterations in cell mechanical properties were caused by activation of TGFβ/Smad signaling pathway in response to TGFβ-stimulation, immunoblotting of Smad2/3 in H1975, H1650, H2030 cell samples was performed, see Fig. 3. As one can see in Fig. 3A, elevation of phosphorylated Smad2/3 was observed in all cell samples reaching the maximum 60 min after TGFβ-stimulation. Treatment of H1975 cells with TGFβR inhibitor SB-431542 completely abolished Smad2/3 phosphorylation (Fig. 3B) as well as the effect of cell stiffening (Fig. 3C) upon treatment with TGFβ.

**Figure 4:**
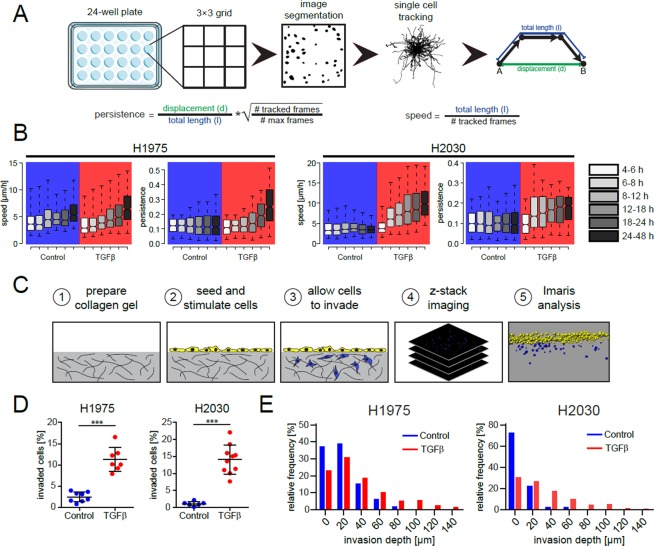
TGFβ-treatment results in the increase of migratory and invasive properties of NSCLC cells. (**A**) Schematic representation of 2D *in-vitro* cell migration assay and parameters describing the migration phenotype. (**B**) Time-resolved effects of TGFβ-stimulation on migration speed and persistence of H1975 and H2030 cells. Center lines show the medians; box limits indicate the 25th and 75th percentiles; whiskers extend to 5th and 95th percentiles. The notches are defined as ±1.58 IQR/$\sqrt{n}$ and represent the 95% confidence interval for each median. At least 300 cells per box from three biological replicates were used. (**C**) Experimental setup of 3D cell invasion in collagen gel. (**D**) Percentage of invaded cells of unstimulated and TGFβ-treated cells after 3 days. (**E**) Frequency diagrams of invasion depth of invaded cells. ***p < 0.001.

Screening for phenotype effects of TGFβ-stimulation was complemented by measurements of spontaneous 2D migration and 3D matrix invasion in H1975 and H2030 cells, see Fig. 4. Thereby, canonical parameters of cell motility and invasion including cell speed, persistence and invasion depth were derived from analysis of 2D and 3D image time series, Fig. 4A,C. Our experimental results showed significant elevation of cell migratory and invasive activity in both NSCLC cell lines, Fig. 4B,D.

**Figure 5:**
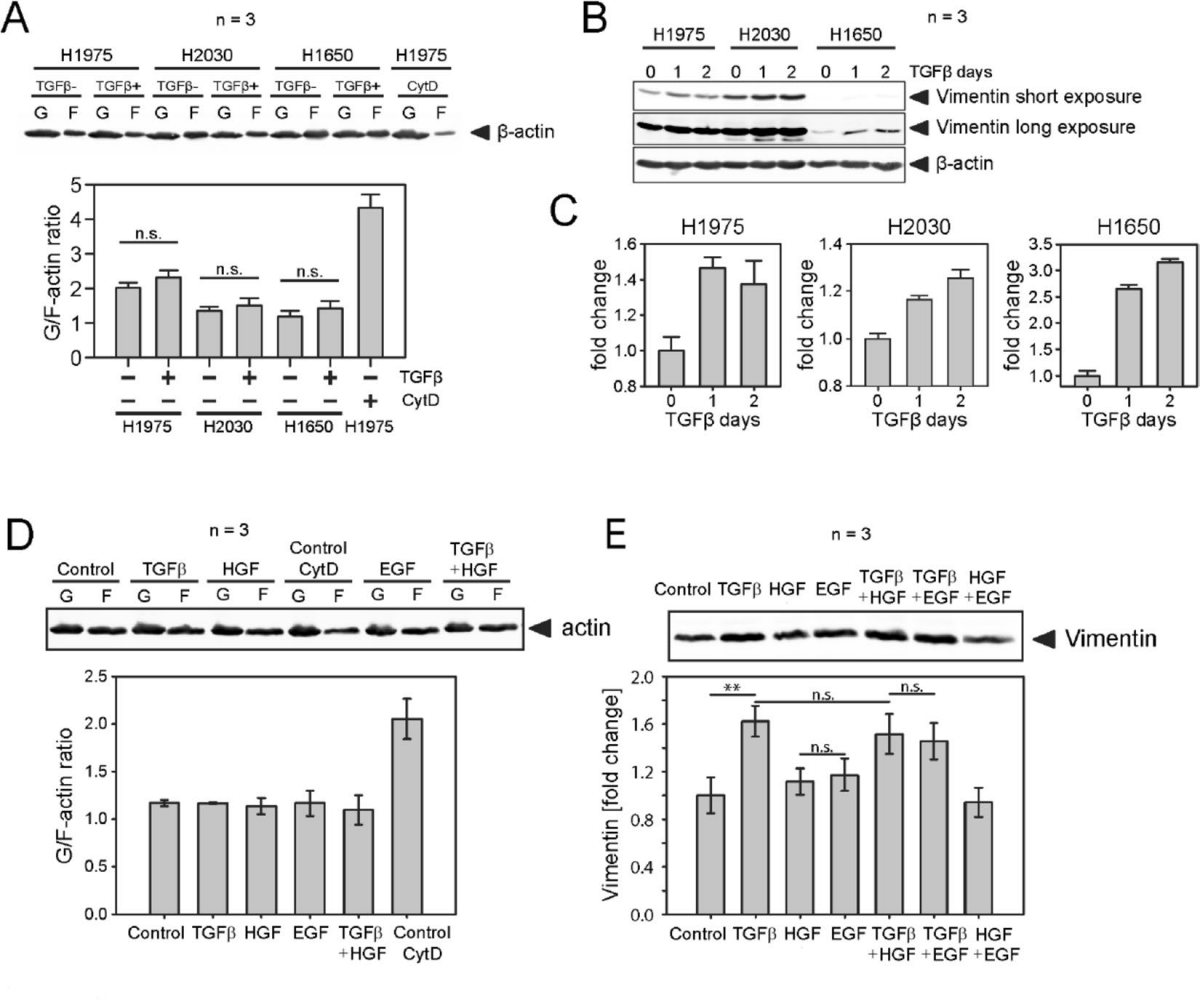
TGFβ-treatment does not change G/F-actin ratio in NSCLC cells, but rather results in increase of vimentin amount. (**A**) G/F-actin ratio in H1975, H1650 and H2030 cells upon treatment with TGFβ was assessed by immunoblot. Equal fractions of G- and F-actin pools were compared. Untreated cells exposed to 20 μM of CytochalazinD for 3 h were used as experimental control. (**B**,**C**) Representative immunoblot of vimentin expression in H1975, H1650 and H2030 cells treated with 2 ng/ml TGFβ for 24 h and respective quantification. (**D**) H1975 cells were treated with 2 ng/ml TGFβ, or 80 ng/ml HGF, or 5 ng/ml EGF, or combination of TGFβ and HGF, or left untreated for 24 h in growth-factor depleted medium. G/F-actin ratio in H1975 cells upon treatment with different growth factors was assessed by immunoblot. Untreated cells exposed to 20 μM of CytochalazinD for 3 h were used as experimental control. (**E**) Vimentin expression in H1975 cells treated with different growth factors for 24 h vs. untreated control. **p < 0.01 and n.s. indicate significant and non-significant differences according to one-way ANOVA. **n** indicates the number of independent repetitions.

**Figure 6:**
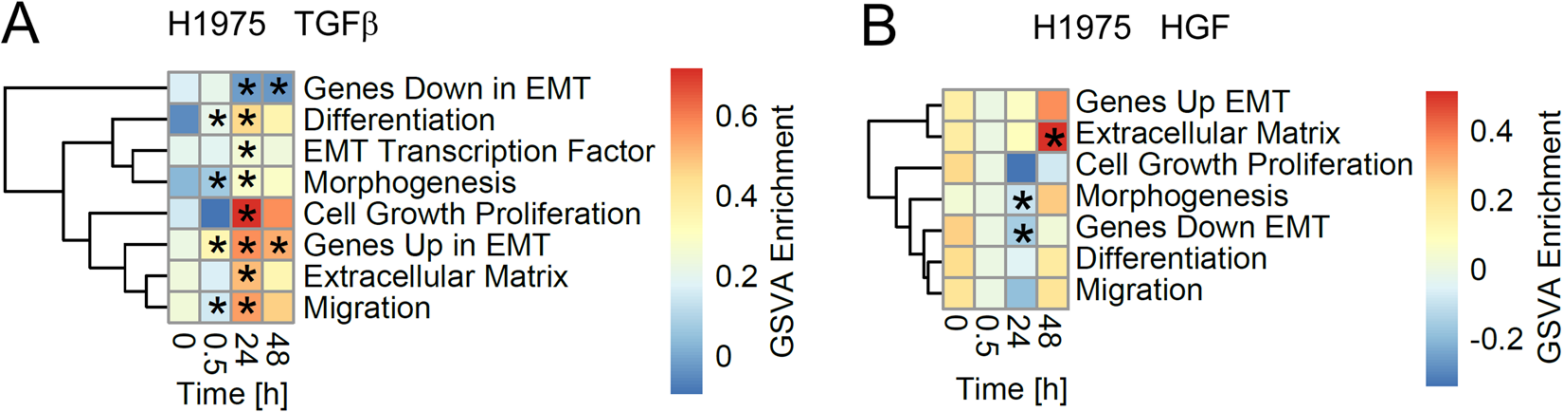
Gene Set Variation Analysis (GSVA) on the longitudinal gene expression data in the H1975 cells upon stimulation with growth factors TGFβ and HGF. The heatmap depicts the average GSVA enrichment scores at 0.5, 24 and 48 hours after TGFβ (**A**) and HGF (**B**) stimulation for time matched (n = 3). Gene sets have been taken from the epithelial-to-mesenchymal transition RT2 Profiler™ PCR Arrays by QIAGEN and are provided as Supplementary Table S1. Stars indicate a significant differential GSVA enrichment score according to a moderated t-test (FDR corrected p-value < 0.05). Rows have been clustered according to their Euclidean distance using complete linkage.

To identify molecular mediators of cell mechanical enhancement upon treatment with TGFβ, we first focused on actin and vimentin filaments found that TGFβ-treatment does not affect the G/F-actin ratio in H1975, H1650 and H2030 NSCLC cells (Fig. 5A), but rather induces overexpression of vimentin, Fig. 5B,C. Additional tests with growth factors HGF and EGF as well as co-stimulation with TGFβ + HGF and TGFβ + EGF left the G/F-actin ratio unaffected, Fig. 5D. Only the EMT-marker vimentin was upregulated in response to TGFβ, Fig. 5E. A Gene Set Variation Analysis (GSVA) on the longitudinal gene expression data confirmed a significant deregulation of further EMT related genes, indicating presence of an epithelial-to-mesenchymal transition in the H1975 cells after TGFβ but not HGF stimulation, see Fig. 6.

In order to systematically assess alterations in gene expression upon stimulation with growth factors on the whole-transcriptome level, mRNA time course experiments with TGFβ- or HGF-treated H1975 cells were performed. An analysis of differential gene expression of TGFβ-stimulated cells (Supplementary Table S2) revealed a significant upregulation of genes associated with actin cytoskeleton, cell migration (leading edge, lamellipodium), myosin complex, and the downregulation of cytokinesis-related genes, see Supplementary Table S3. In comparison to TGFβ, HGF-stimulated cells respond with activation of a significantly smaller number of proteins related to actin cytoskeleton.

To assess a possibly broad range of potential affectors of cell mechanical properties, we created a manually curated set of 812 genes by integrating all GO terms related to cell mechanical components and functions such as actin cytoskeleton, microtubules, intermediate filaments, myosins, cell division, cell migration, cell-cell and cell-ECM junctions, see Supplementary Table S4. We found that 210 of 812 genes (26%, *p* < 0.001 hypergeometric test) were significantly upregulated and 170 (21%, *p* < 0.001 hypergeometric test) downregulated in TGFβ-treated H1975 cells, see Supplementary Table S5. In HGF-stimulated samples, the number of differentially expressed ‘cell mechanics’ genes turned out to be about 10 times smaller (i.e. 23 up- and 17 downregulated). Genes upregulated upon HGF-stimulation are associated with actin and microtubule cytoskeleton and motor activity as well as caveolae, see Supplementary Table S6.

**Figure 7:**
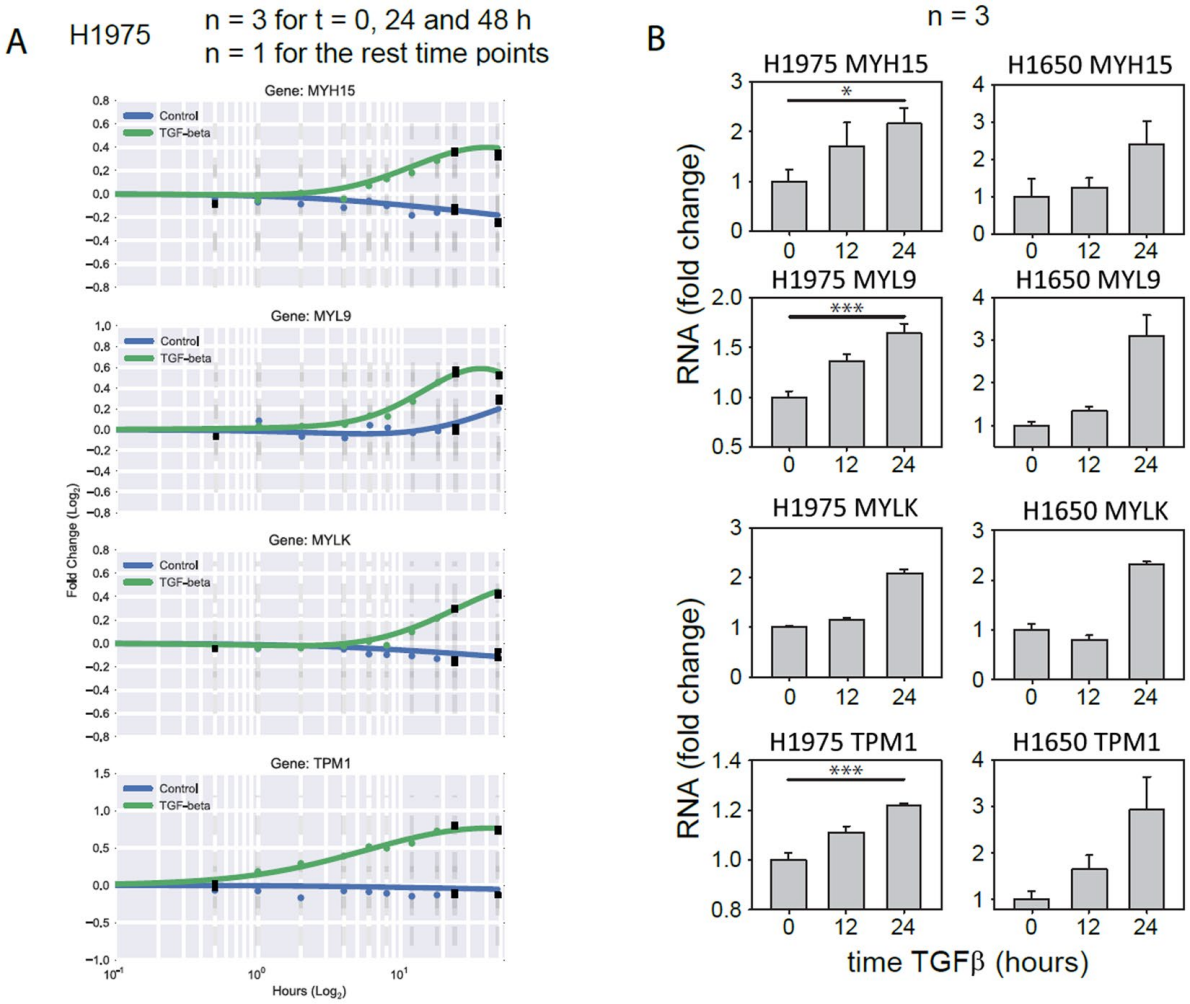
Dynamic response of MYH15, MYL9, MYLK and TPM1 to TGFβ-stimulation in NSCLC cell lines. (**A**) Single gene dynamics of candidates genes from the microarray. (**B**) Validation of the microarray candidate genes with qPCR in H1975 and H1650 cells. Cells were stimulated with 2 ng/ml TGFβ and RNA was extracted at the indicated time points. Gene expression was assessed using qRT-PCR. mRNA expression was normalized to the geometric mean of two housekeeper genes: G6PD and HPRT. *p < 0.05 and ***p < 0.001 significance levels were determined using one-way ANOVA. **n** indicates the number of independent repetitions.

**Figure 8:**
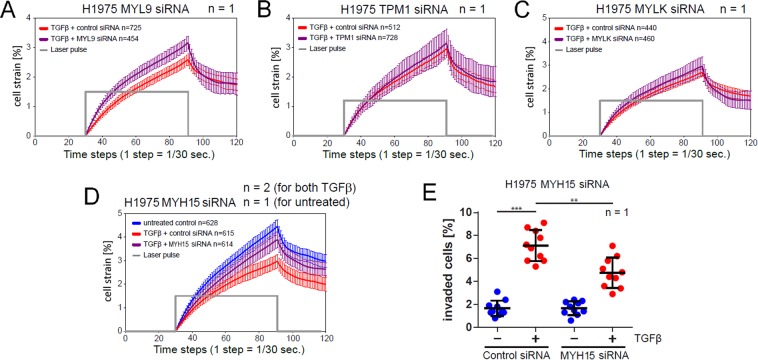
siRNA-mediated knockdown of MYH15 and MYL9, but not MYLK and TPM1 reduces TGFβ-induced stiffening of H1975 cells. (**A**–**D**) Creep-and-recovery curves of H1975 transfected with either non-targeting siRNA or siRNA specific to one of the candidate genes for 36 h followed by stimulation with 2 ng/ml TGFβ for 24 h. Afterwards cells were trypsinized and injected into the microfluidic system of optical stretcher. MYH15 siRNA-silenced H1975 cells exhibit the largest softening in comparison to control probes among four tested candidate genes. (**E**) TGFβ-induced 3D collagen gel invasion of H1975 cells upon siRNA-mediated MYH15 knockdown. Cells were siRNA-transfected and seeded on collagen gels, allowed to adhere overnight and stimulated with 2 ng/ml TGFβ for 3 days. Afterwards cells were fixed, stained with Hoechst and imaged on confocal microscope (Zeiss LSM710). **n** inside the boxes indicates the number of cells measured per condition, **n** outside the boxes corresponds to the number of independent repetitions. **p < 0.01 and ***p < 0.001 significance levels are according to one-way ANOVA.

For evaluation of the phenotype relevance of the above gene candidates, we focused on actin cytoskeleton and actomyosin associated proteins that are mechanistically related to cell rigidity and motility, and excluded genes encoding extracellular and membrane proteins. Four following genes upregulated in TGFβ-treated samples were selected for a closer analysis: MYH15, MYL9, MYLK, TPM1. Figure 7 shows they dynamic response of these genes to stimulation with TGFβ over 48 hours as measured from the microarray (Fig. 7A) and validated by RT-qPCR (Fig. 7B). MOS measurements under siRNA-mediated knockdown of MYH15 and MYL9 but not MYLK and TPM1 lead to a reduction of TGFβ-induced stiffening of H1975 cells, Fig. 8A–D. Reduced stiffening was, furthermore, accompanied by decreased invasiveness of H1975 cells. Measurement of TGFβ-induced 3D collagen gel invasion of H1975 cells showed a significant slowdown of invasive cell activity upon siRNA-mediated MYH15 silencing, Fig. 8E.

## Discussion

Here we performed mechanical phenotyping and transcriptome analysis of NSCLC cells with the goal to dissect cytoskeletal mediators of increased cell stiffness and migratory activity upon stimulation with the growth factor TGFβ complemented by comparative stimulation with HGF. Our experimental results show that (i) TGFβ stimulation elevates rigidity, size, motility and invasiveness of NSCLC cells, and that (ii) these changes in cell mechanical phenotype are accompanied by upregulation of vimentin intermediate filaments, adhesion/migration related and unconventional motor proteins.

In the last two decades, material properties of cancer cells were in focus of numerous previous studies leading to the general notion that cells undergoing malignant and metastatic transformations become progressively softer^29–31^. From this perspective, our observation of NSCLC cell stiffening upon GF-stimulation, which is believed to trigger acquisition of metastatic phenotype, is contradictory to the prevailing view on cell rigidity changes during metastatic transformation. Recent findings by Tavares *et al*.^32^ show, however, that cancer cells can also undergo temporal stiffening within the initial phase of metastatic transformation. There are several possible explanations for this apparent contradiction. First, previous comparisons between rigidity of normal, malignant and metastatic cells were typically performed without growth factors stimulation. Our *in-vitro* GF-stimulation experiments may be akin to physiological conditions that cancer cells experience *in-vivo* at the invasive tumor margin, having the highest concentration of growth factors and mesenchymal markers^33^. Biopsy samples with primary tumor cells are, however, typically obtained from the central tumor region, where cells are exposed to microenvironmental conditions considerably different as compared to the tumor margin^34^. Cell mechanical properties and migratory behavior are known to be controlled by a tight interplay between environmental cues and cell sensory pathways such as Rac1/RhoA GTPase-based circuits that mediate amoeboid-to-mesenchymal transition^3,35^. Reliable data on differences in cell mechanical properties of amoeboid and mesenchymal phenotypes are presently missing. Our findings suggest that stimulation with growth factors induces mesenchymal (TGFβ) or mixed (HGF) phenotypes of NSCLC cells that appear to differ from a pure amoeboid one not only by cell morphology and type of migration^36^, but also by mechanical rigidity. After leaving the microenvironment of the tumor margin, cancer cells invading normal tissue are exposed to further chemical and physical conditions that may trigger the reversal of their migration mode and mechanical properties by means of the mesenchymal-to-amoeboid transition^3,37,38^. Sensitivity of cells, not only to chemical, but also to physical environment means that measurements of cell mechanical properties using different assays and protocols (i.e., culturing cells on soft vs stiff substrates, probing adherent cells with AFM vs probing soluble cells with MOS) may, in general, lead to contradicting results^39^. Consequently, further investigations are required to generalize findings acquired with our particular experimental set-up.

The results of our differential gene expression analysis show large-scale reorganization of cytoskeletal architecture and signaling landscape in TGFβ-stimulated NSCLC cells. Our observations of elevated stiffness and migratory activity of TGFβ-treated H1975, H1650, H2030 NSCLC cells are in good agreement with previous findings in other NSCLC cell lines^40^. Overexpression of vimentin as well as cell adhesion/migration relevant proteins (MYLK, MYL9, TPM1) selected for exemplary phenotype-genotype correlation in this study was frequently observed in different cell lines upon EMT or malignant transformation. MYLK was implicated in mediation of transcellular intravasation of breast cancer cells^41^, influencing global gene regulation in cancer^42^. High level of MYL9 expression was observed in injury, aging^43^ and esophageal squamous cell carcinoma^44^. TPM1 overexpression was found in NSCLC and neuroblastoma^45^ and implicated in modulation of focal adhesion and cell migration behavior^46,47^. MYH15 myosin was selected in this study for a closer analysis because of potential involvement of non-muscle class II myosins in tumor progression, cancer cell invasion, metastasis^48,49^ and EMT^50^. Hansel *et al*.^51^ report upregulation of MYH15 by the airway epithelium, vascular endothelium, and inflammatory cells in patients with chronical pulmonary diseases (COPD) exhibiting elevated airway responsiveness and inflammation. Our experiments with gene-silenced cells revealed that MYH15 and MYL9, but not MYLK and TPM1 affect cell rigidity. We trace this result back to the functional role of MYH15 and MYL9 as actin crosslinkers enhancing effective stiffness of actin cytoskeleton network. Knockdown of MYH15 was further shown to significantly reduce invasiveness of H1975 cells which indicates an active functional role of this unconventional myosin in H1975 NSCLC cells. Thus, NSCLC cells respond to TGFβ-stimulation with upregulation of several cancer type unspecific (VIM, MYLK, MYL9, TPM1) but also more lung disease specific (MYH15) proteins.

Comparative measurements with HGF and HGF + TGFβ co-stimulated NSCLC cells demonstrate specificity of phenotypic and transcriptomic effects triggered by TGFβ that can be, however, modulated by co-treatment with other growth factors. In contrast to TGFβ, a significantly smaller number of actin and microtubule cytoskeleton associated genes are differentially expressed in response to HGF-stimulation. An interesting point for future investigations of HGF effects on cell mechanics is upregulation of caveolins that were previously linked to regulation of cell stiffness and mechanosensing^31,52^. Finally, it should be mentioned that optical deformability used in this study for characterization of cell rigidity is a complex physical property which depends not only on material stiffness but also on cell optical properties such as refractive index and total light-interception area (i.e. cell size)^53,54^. Consequently, our optical deformability measurements can be related to cell mechanical stiffness only under assumption of otherwise equal optical properties of GF-treated and -untreated cells. On the other hand, increase of cellular and nuclear size of GF-stimulated cells, which probably results from their enhanced metabolic activity^55^, should lead to effectively larger optical forces and cell deformations. This was, however, not the case. In contrast, enlarged GF-treated cells exhibit reduced deformability which means that after normalization by the cell size the actual increase of cell stiffness after GF-stimulation is even higher.

In summary, our results provide novel insights into phenotypic and transcriptomic response of NSCLC cells to stimulation with TGFβ and suggest that mediators of elevated cell stiffness and migration activity such as overexpressed vimentin intermediate filaments, components of actin and microtubule cytoskeleton, actomyosin complex, and, in particular, unconventional myosins represent promising pharmaceutical targets for suppressing metastatic dissemination of lung cancer cells.

## Methods

### Cell lines and culture conditions

Human lung adenocarcinoma cell lines H1975, H2030 and H1650 were purchased from ATCC and cultivated in Dulbecco’s modified Eagle’s Medium (DMEM, Lonza) supplemented with 10% FCS (Gibco) and 1% penicillin/streptomycin (Gibco). All cell lines were authenticated. Prior to stimulation with growth factors, cells were kept overnight in growth factor depleted medium supplemented with 1 mg/ml BSA if not stated overwise. 2 ng/ml of recombinant human TGFβ (RD Systems #240-B-010) and 80 ng/ml of recombinant human HGF (R D Systems #294-HG-025) were used for stimulation NSCLC cells as suggested in the literature^56,57^.

Primary tumor cells were provided by the Lung Biobank Heidelberg, a member of the accredited Tissue Bank of the National Center for Tumor Diseases (NCT) Heidelberg, the BioMaterialBank Heidelberg, and the Biobank platform of the German Center for Lung Research (DZL). All patients provided written informed consent for the use of their biomaterials for research purpose. The studies were approved by the local ethics committee of the University of Heidelberg (No. 270/2001). All methods were performed in accordance with the relevant guidelines and regulations. Isolation and cultivation of primary cells from NSCLC tissues were performed according to^58,59^. In brief, tumor tissues were gently minced in bovine serum albumin (BSA, 10 mg/ml, PAA) containing DMEM/Ham’s F12 (1:1) medium (Life Technologies). The resulting cell suspension was centrifuged on Histopaque-1077 (Sigma-Aldrich). Collected cells from the interphase were grown on collagen coated flasks in serum-free ACL-4 medium^59^ containing 10 μM ROCK inhibitor (ENZO Life Sciences). A summary of two patient samples is in Table 1.

**Table 1:** Summary of primary NSCLC samples.

Sample No.	Gender	Age	Histology	p-Stage	Smoker status
4950T	female	59	adenocarc.	IIIa	smoker
5297T	male	66	adenocarc.	Ia	former smoker (6 month)

### Microfluidic optical stretcher measurements of cell deformability

To quantitatively assess the effects of TGFβ-treatment on cell deformability in a high-throughput manner, NSCLC cells were stimulated with TGFβ for 24 h in FCS-free medium, detached with trypsin/EDTA solution (Gibco), cleared from debris by centrifugation and resuspended in PhenolRed-free DMEM to a concentration of 600,000 cells/ml and injected into the microfluidic system^60,61^. The measurement principle of MOS is based on exposure of soluble cells to two opposing rays of infrared laser light (wavelength = 750 nm), which induces continuous uniaxial stretching of the cell along the laser axis. The measurement of each cell is done in three time steps: monitoring of the unloaded cell for one second followed by two seconds of laser-induced cell stretching (creep phase) and, finally, monitoring after application of the laser pulse for one second (recovery phase). The optical appearance of cells is captured in time-series of 120 images in total that are subsequently processed to quantify cell contour changes under the impact of optical stretching forces. In particular, for every time step of the image sequence the diameter of the stretched cell axis *L*(*t*) is determined. On the basis of smoothed time series *L*(*t*), the dynamic strain response of the cell $\varepsilon (t)$ is computed as ${\varepsilon }_{i}(t)={L}_{i}(t)$/${L}_{i}(0)-1$, where *L*_*i*_(*t*) denotes the diameter of the stretched axis of the *i*-th cell. Since the type of the statistical distribution of cell compliance is a priori unknown and the cell-to-cell variability is relatively high, a parameter-free bootstrap approach is applied to calculate the empirical distribution of sample means, their average values ${\varepsilon }_{bs}(t)$ and corresponding confident intervals, i.e., two-fold standard deviation of bootstrap mean statistics^62^. The key parameter used for characterization of the rigidity of cell samples in this work is the maximum strain value of the entire bootstrap-averaged creep-and-recovery curve which is typically achieved by the end of the creep phase, i.e., ${\varepsilon }_{{\rm{\max }}}=\,{\rm{\max }}\,({\varepsilon }_{{\rm{bs}}}(t))$.

### Immunoblotting

Cells were lysed in RIPA buffer (50 mM Tris pH 7.4, 150 mM NaCl, 1 mM EDTA (AppliChem), 10 mM NaF, 1% (v/v) NP40, 0.1% sodium deoxycholate, 2 μg/ml aprotinin and 200 μg/ml AEBSF). Cell extracts were cleared by centrifugation at 18,000 g for 10 min at 4 °C and the protein concentration of each sample was measured by the BCA protein assay (Pierce). Equal amount of lysates were subjected to 10% SDS – PAGE and subsequently transferred to nitrocellulose membrane (Millipore). Blots were blocked with blocking buffer for infrared immunoblotting (LI-COR #927-40000) for 1 h and co-incubated with primary antibodies against vimentin (Cell Signaling #5741) and actin (Sigma Aldrich #A5441) overnight at 4 °C. Secondary antibodies coupled to IRDye infrared dyes (LI-COR #926-32211 and #926-68070) were used for detection with infrared Odyssey imager (LI-COR). Signal quantification was performed using an ImageQuant system (GE Healthcare). Replicates from different membranes were scaled and averaged using methods described in^63^. Vimentin blot images were acquired with both short and long exposure times. Thereby, no significant dependency of vimentin expression measurements on duration of exposure was observed.

### Measurement of G-actin/F-actin ratio

G/F-actin ratio was determined as described in (Rasmussen *et al*.^64^). Briefly, cells were lysed in actin stabilization buffer (0.1 M PIPES, pH 6.9, 30% glycerol, 5% DMSO, 1 mM MgSO4, 1 mM EGTA, 1% TritonX-100, 1 mM ATP, 2 μg/ml aprotinin and 200 μg/ml AEBSF) on ice for 10 minutes. Cells were harvested and the cell extracts were centrifuged at 4 °C for 75 minutes at 16,000 g to separate F- and G-actin pools. The supernatants of the extracts were collected and designated a G-actin pool. The pellets were resuspended in ice-cold actin depolymerization buffer (0.1 M PIPES, pH 6.9, 1 mM MgSO4, 10 mM CaCl2, and 5 μM cytochalasin D) and designated as F-actin pool. Equal amounts of both the supernatant (G-actin) and the resuspended pellet (F-actin) were subjected to Western blot with the use of an anti-β-actin antibody (Sigma Aldrich #A5441).

### RNA expression analysis with quantitative real-time PCR (qRT-PCR)

Total RNA was extracted using RNeasy Plus Mini Kit (Qiagen) according to the manufacturer’s instructions. RNA was eluted in 30 μl of RNAse free water and stored at −80 °C. Concentration of total RNA was measured on NanoDrop (Thermo Scientific). From each RNA sample two reverse transcription reactions were performed in a volume of 20 μl from 1.5 μg RNA with High Capacity cDNA Reverse Transcription Kit (Applied Biosys-tems) with a program recommended by manufacturer. Later both reactions were pulled together. qRT-PCR was performed using a LightCycler 480 (Roche Diagnostics) in combination with Universal ProbeLibrary (UPL) platform (Roche Diagnostics). Primers for UPL assays were generated using the online UPL Assay Design Center (http://www.roche-applied-science.com/sis/rtpcr/upl), which also suggests an optimal probe for a given replicon. Primers were purchased from Eurofin MWG. qRT-PCR amplifications were performed in 384-well plates containing 6 μl of a master mix (Roche Diagnostics) and 5 μl of diluted cDNA. For pippeting of the master mix and the appropriate amounts of cDNA onto 384-well plate, the pipetting robot MICROLAB® STARlet (Hamilton) was used, following an in house developed pipetting protocol. A following PCR program was used for cDNA amplification. For analysis, the Second Derivative Maximum method of the LightCycler 480 Basic Software (Roche Diagnostics) was used to calculate crossing point values. Dilution series of template cDNA were used to correct PCR efficiency for each pair of primers in each cell line. Relative concentrations were normalized to a geometric mean of housekeeper genes.

### Gene expression array

To analyze the gene expression pattern in H1975 after TGFβ-stimulation, a mRNA time course experiment with different time points t = [0 h, 0.5 h, 1 h, 2 h, 4 h, 6 h, 8 h, 12 h, 18 h, 24 h, 48 h] was performed and analyzed by a mRNA microarray (GeneChip 2.0 ST, Affimetrix). One replicate of each time point was used for the analysis except of the time points 0, 24 and 48 hours which were measured in triplicates. Microarray data has been deposited at the Gene Expression Omnibus (GEO) database under the access ID GSE98979 and is accessible to the reviewers using the token qnifsmskxtkffyd.

### Microarray data analysis

Gene expression data from the Affymetrix microarray experiments were used. The raw data were normalized via RMA^65^ and converted to fold changes with respect to t = 0 h. Replicates were mean-averaged and inconsistencies between the control and stimulus time-courses were linearly interpolated. For smoothing of gene expression time-series, Gaussian Process regression^66^ was applied. Entrez and gene symbol annotations were done via biomaRt^67^. Gene ontology (GO) enrichment of differentially expressed gene sets was performed using GAGE^68^ and GSVA^69^ with the default parameter settings and the cut-off threshold *p* < 0.05 for FDR corrected p-value of t-test^70^. The EMT-related gene sets are based on the EMT RT2 Profiler™ PCR Arrays by QIAGEN, see Supplementary Table S1.

### Statistical analysis

SigmaPlot (Systat Software), GraphPad Prism (GraphPad Software) and BoxPlotR^71^ were used for plotting and representing data. All numeric data are presented as mean SEM or SD from at least three biological replicates. For all statistical analysis, ANOVA or, where appropriate, unpaired t-test was used. Results were considered significant at *p* < 0.05.

### *In-vitro* 2D cell migration assay

For 2D migration assay, H1975 cells were seeded in 24-well plate (Zell Kontakt # 3231-20) at a density of 6000 cells/well. Cells were allowed to attach for 18 μh, serum-starved for 3 μh, stained with Hoechst (Sigma) for 1 μh, stimulated with growth factors and imaged on environment-controlled microscope (IX81, Olympus). Images were acquired with an UPlanSApo 10×/0.4 objective lens (Olympus) in 30 μmin intervals for 48 μh. Nine positions per well (3 × 3 grid) were imaged and stitched with ImageJ plugin^72^. Single cell tracking was performed with ImageJ Mtrack2 plugin. Speed of each tracked cell was calculated by dividing total traveled distance by total time, for which cell was tracked. Persistence was calculated by dividing the distance between the first and the last point, where the cell was tracked, by total travelled distance. Resulting number was multiplied by the square root of time, for which cell was tracked divided by maximal possible tracking time, in order to penalize cells, which were tracked for a shorter period of time.

### 3D collagen invasion assay

3D collage gels were prepared as described previously^73,74^. Briefly, 50 μl of collagen G solution (L1613, Biochrome) mixed with ice-cold 1 M HEPES buffer, 0.7 M NaOH, 10× PBS pH 8.0 was added per well of 96-well plate with a flat bottom (BD #353376). A plate was kept overnight at 4 °C and then for least 1 h at 37 °C to allow gelation of the collagen. 7500 cells/well were seeded on top of the matrix and allowed to attach overnight. Cells were stimulated with 2 ng/ml TGFβ in serum-deprived medium with 1 mg/ml BSA and left for invasion for 3 days. Afterwards, cells were fixed in 3.7% PFA for one hour and subsequently stained with 1:1000 Hoechst (Sigma) in PBS for one hour. 2 × 2 tile z-stacks were acquired for each well using LSM710 confocal microscope (Carl Zeiss) equipped with EC Plan-Neofluar DIC 10×/0.3 NA objective lens (Carl Zeiss). Images were analyzed using Imaris software (Bitplane). The fluorescent intensity of each nucleus was represented by a spot using spots detection algorithm. In order to distinguish invaded cells from the cells on top of the collagen matrix, these spots were thresholded by their z-position. As output the percentage of invaded cells and the invasion depth was obtained.

### Fluorescent microscopy

H1975 cells were seeded 7500 cells per well in 24-well plates (Zell Kontakt # 3231-20) with full DMEM containing no Phenol red (Lonza). Cells were allowed to attach for 24 hours and were then stained stimulated with respective growth factors or left untreated for 24 h in serum-deprived medium with 1 mg/ml bovine serum albumin (BSA). Afterwards cells were stained with 1:1000 diluted Hoechst (Sigma) for one hour and imaged using automated microscope (IX81, Olympus) with an UPlanSApo 10×/0.4 objective lens (Olympus). Acquired images were processed and segmented in ImageJ. Stitching was performed using ImageJ ‘Stitch Sequence of Grid of Images’ plugin^72^. ImageJ (NIH, Bethesda) was used to quantify the cell nucleus size.

## Supplementary information

Supplementary Information

Supplementary Table S1

Supplementary Table S2

Supplementary Table S3

Supplementary Table S4

Supplementary Table S5

Supplementary Table S6

## Data Availability

In addition to data presented in the main text, microarray data has been deposited at the Gene Expression Omnibus (GEO) database under the access ID GSE98979.
