# Supplementary material for: TGFβ-induced cytoskeletal remodeling mediates elevation of cell stiffness and invasiveness in NSCLC
Source: Sci Rep. 2019 May 21;9:7667. doi: 10.1038/s41598-019-43409-x (PMC6529472; doi:10.1038/s41598-019-43409-x)

Supplementary Information

Article in *Scientific Reports*

TGFβ-induced cytoskeletal remodeling mediates elevation of cell stiffness and invasiveness in NSCLC

E. Gladilin, S. Ohse, M. Boerries, H. Busch, C. Xu, M. Schneider, M. Meister, R. Eils

Supplementary Table S1. Gene sets used for Gene Set Variation Analysis (GSVA) on the longitudinal gene expression have been taken from the epithelial-to-mesenchymal transition RT2 Profiler™ PCR Arrays by QIAGEN. (see Excel file)

Supplementary Table S2. Differential gene expression analysis (whole genome scale) of the non-small cell lung cancer cell line H1975 24h after treatment with TGFβ or HGF. (see Excel file)

Supplementary Table S3. Ontology enrichment of differentially expressed genes in the non-small cell lung cancer cell line H1975 24h after treatment with TGFβ or HGF. (see Excel file)

Supplementary Table S4. Manually curated set of 812 genes related to cell mechanical components and functions. (see Excel file)

Supplementary Table S5. Differential gene expression of cell mechanics related genes in the non-small cell lung cancer cell line H1975 24h after treatment with TGFβ or HGF. (see Excel file)

Supplementary Table S6. Ontology enrichment of differentially expressed cell mechanics related genes in the non-small cell lung cancer cell line H1975 24h after treatment with TGFβ or HGF. (see Excel file)

Supplementary Figure S1. Full length blot images of cropped western blots. (A) Figure 3A, (B) Figure 3B.

(A)

H1975

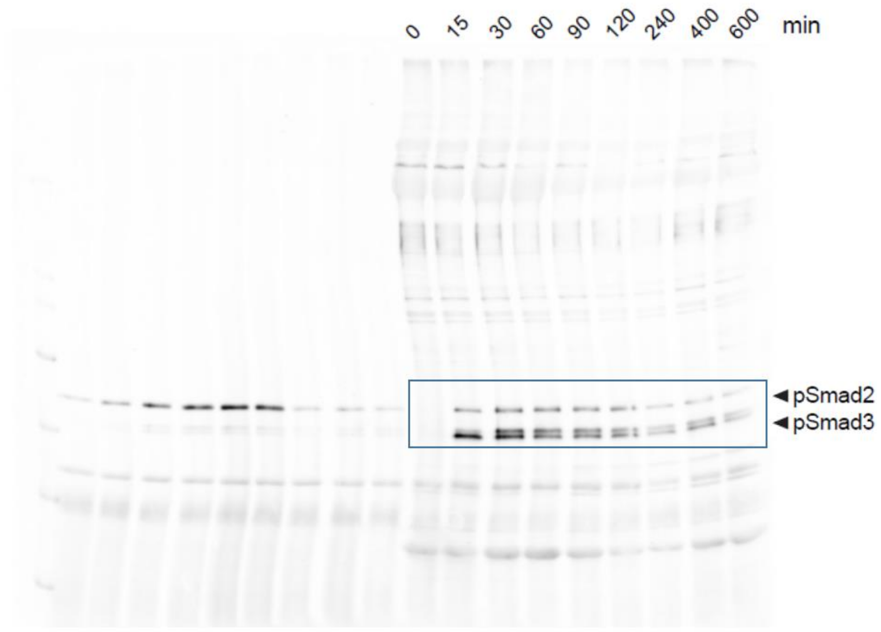

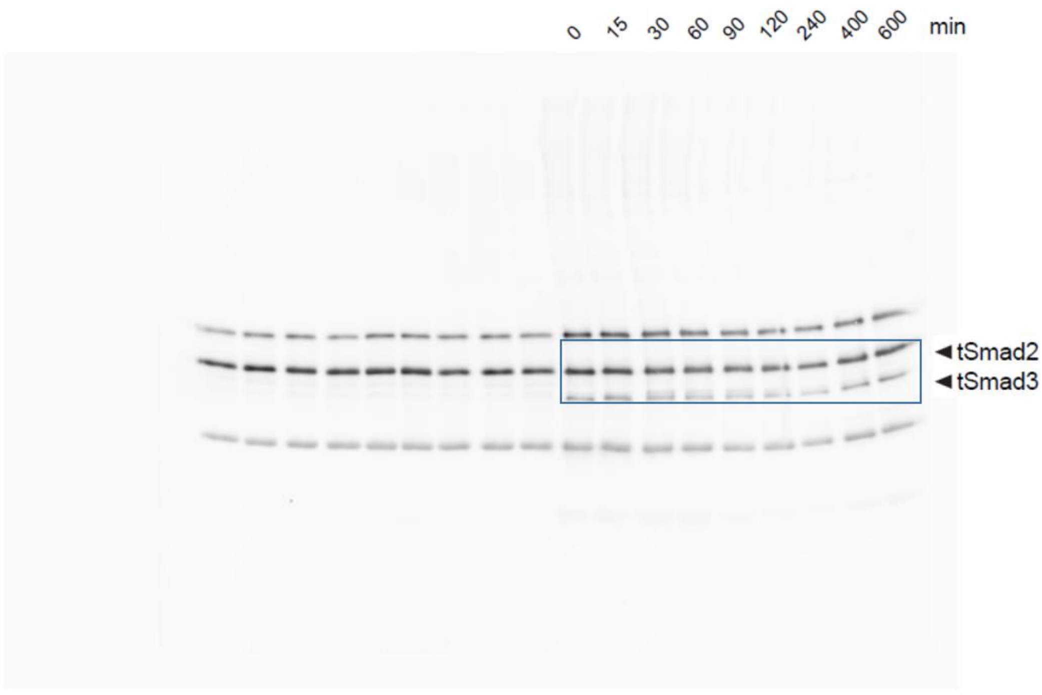

H1650

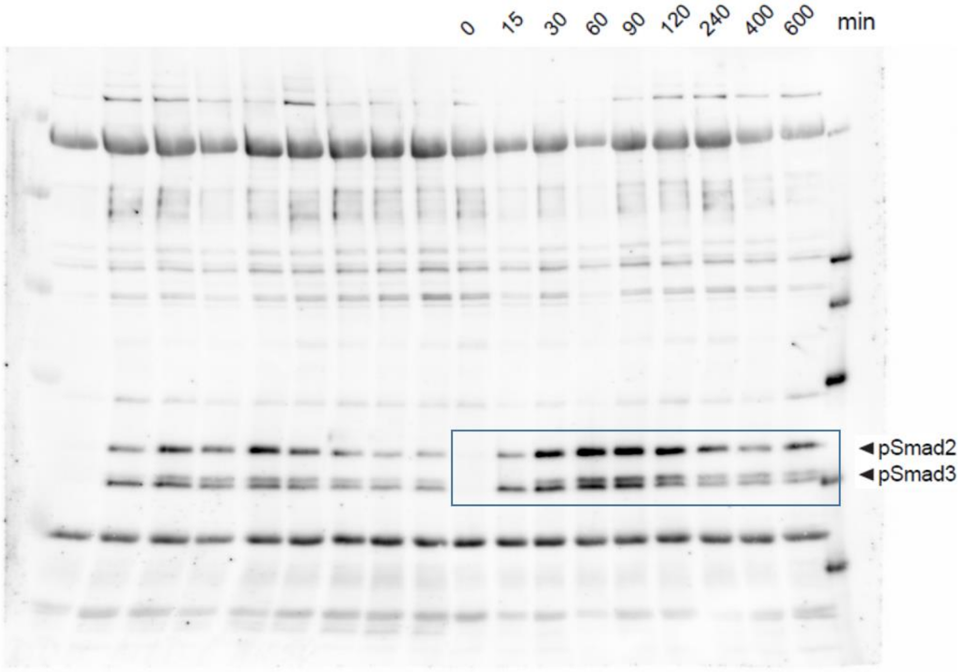

0 15 30 60 90 120 240 400 600 min
pSmad2
pSmad3

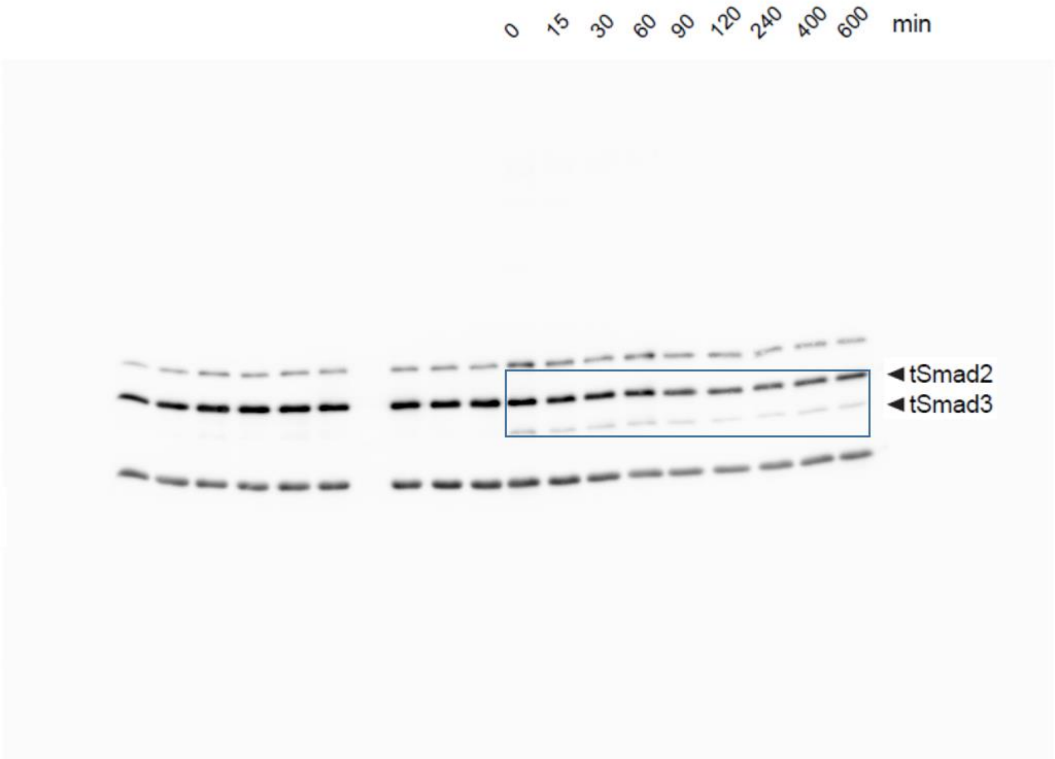

0 15 30 60 90 120 240 400 600 min
tSmad2
tSmad3

H2030

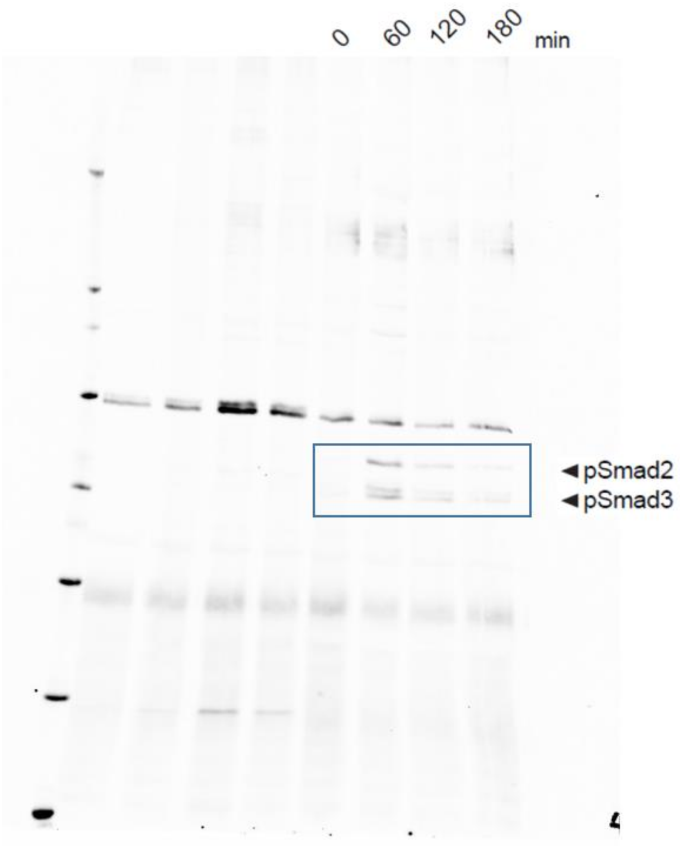

(B)

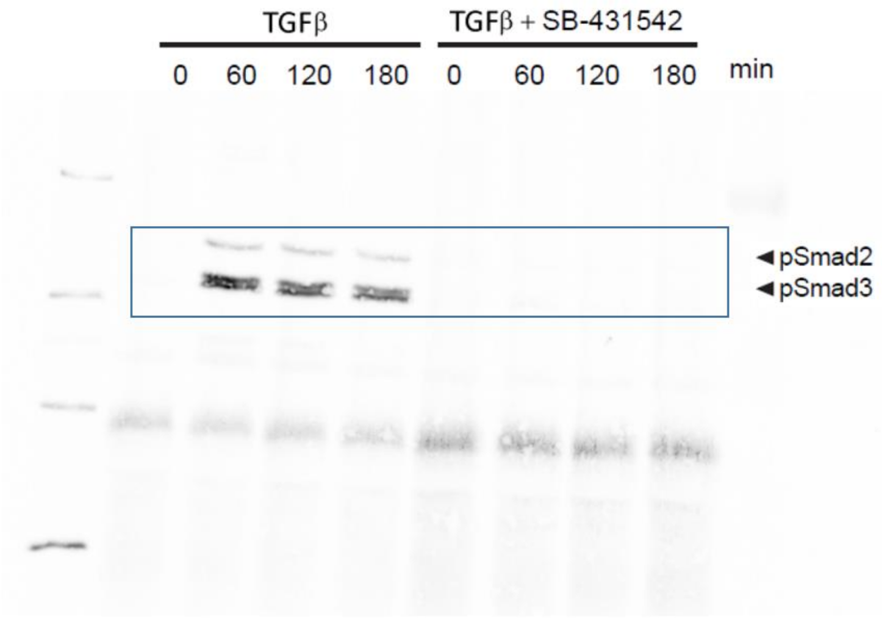

TGFβ
TGFβ + SB-431542
0 60 120 180 0 60 120 180 min
◄pSmad2
◄pSmad3

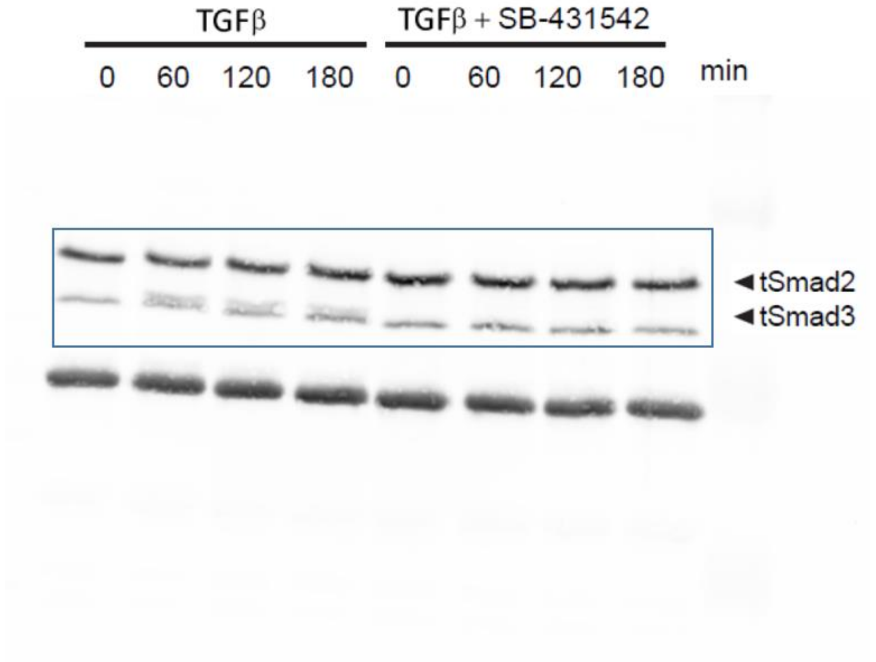

TGFβ
TGFβ + SB-431542
0 60 120 180 0 60 120 180 min
◄tSmad2
◄tSmad3

Supplementary Figure S2. Full length blot images of cropped western blots. (A) Figure 5A, (B) Figure 5B, (C) Figure 5E.

(A)

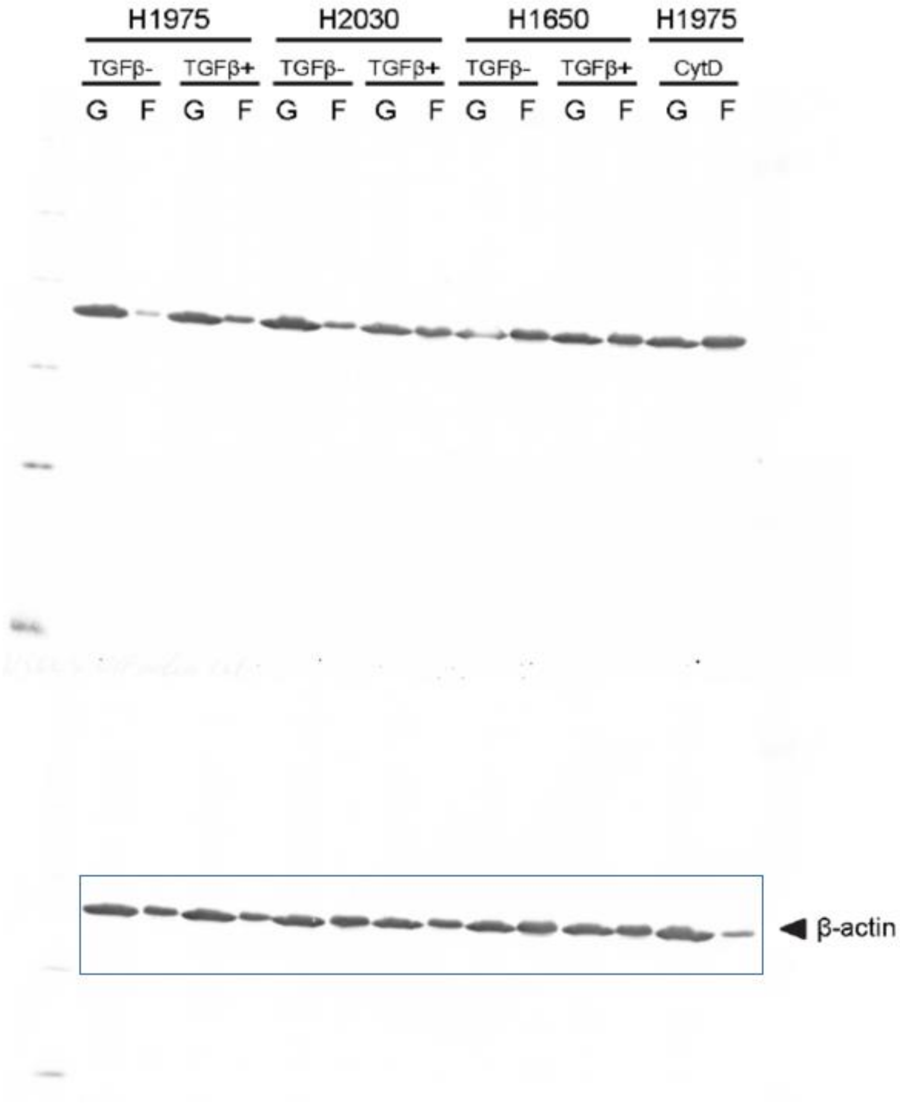

(B)

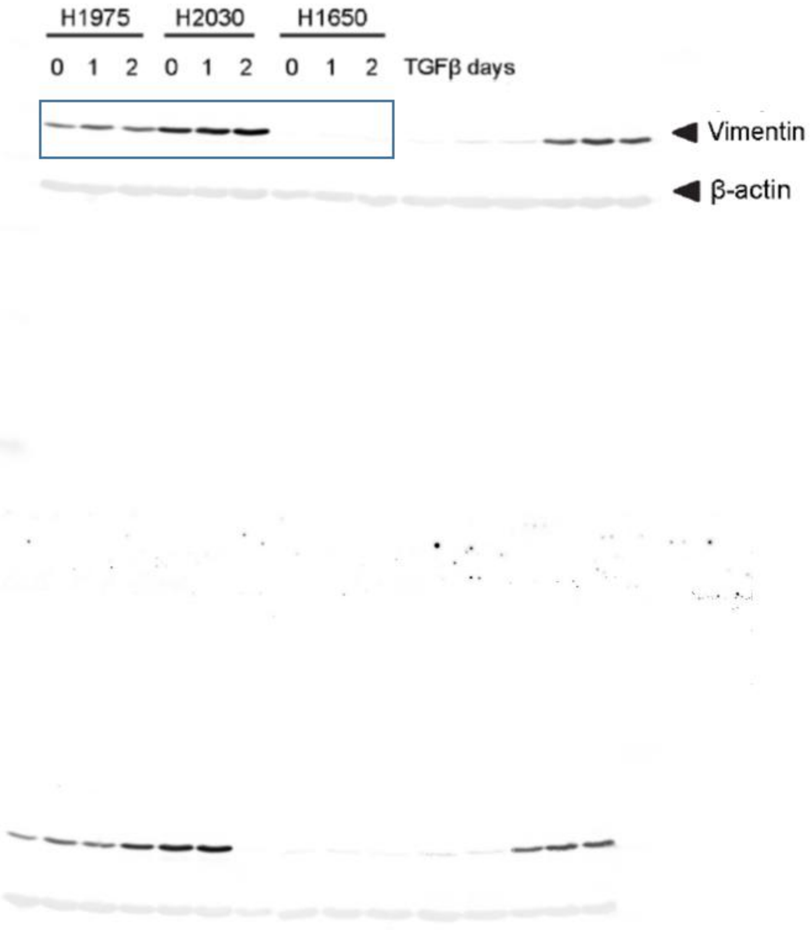

(C)

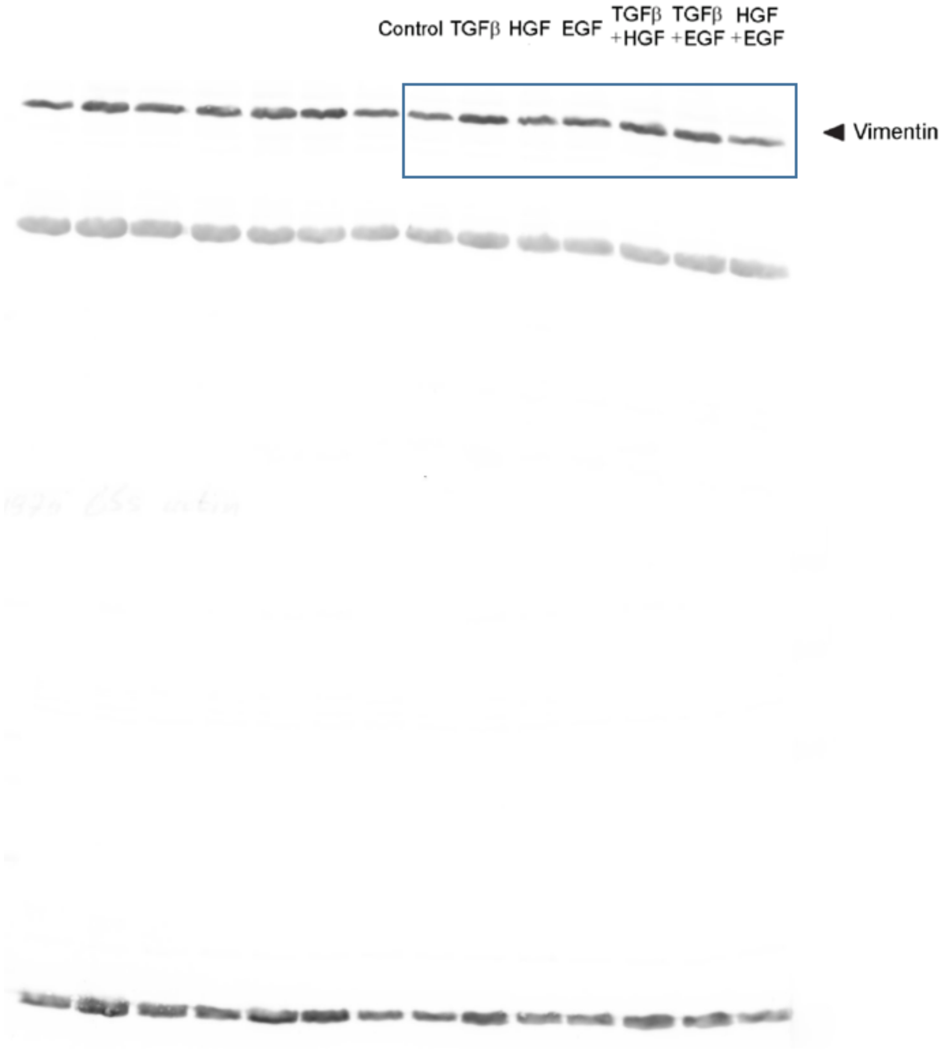

Supplement: Supplementary file 1 — Supplementary Information [file 41598_2019_43409_MOESM1_ESM.pdf]
